# A multiplex immunoassay for the non-invasive detection of bladder cancer

**DOI:** 10.1186/s12967-016-0783-2

**Published:** 2016-01-30

**Authors:** Yoshiko Shimizu, Hideki Furuya, Peter Bryant Greenwood, Owen Chan, Yunfeng Dai, Mark D. Thornquist, Steve Goodison, Charles J. Rosser

**Affiliations:** University of Hawaii Cancer Center, 701 Ilalo St, Rm 327, Honolulu, HI 96813 USA; Department of Molecular Biosciences and Bioengineering, University of Hawaii at Manoa, Honolulu, HI USA; Department of Biostatistics, University of Florida, Gainesville, FL USA; Cancer Prevention Program, Fred Hutchinson Cancer Research Center, Seattle, WA USA; Nonagen BioScience Corp, Jacksonville, FL USA; Department of Health Sciences Research, Mayo Clinic, Jacksonville, FL USA

**Keywords:** Biomarkers, Bladder cancer, Multiplex, Protein, Urine

## Abstract

**Background:**

Urine based assays that can non-invasively detect bladder cancer (BCa) have the potential to reduce unnecessary and invasive procedures. The purpose of this study was to develop a multiplex immunoassay that can accurately and simultaneously monitor ten diagnostic urinary protein biomarkers for application as a non-invasive test for BCa detection.

**Methods:**

A custom electrochemiluminescent multiplex assay was constructed (Meso Scale Diagnostics, LLC, Rockville, MD, USA) to detect the following urinary proteins; IL8, MMP9, MMP10, ANG, APOE, SDC1, A1AT, PAI1, CA9 and VEGFA. Voided urine samples from two cohorts were collected prior to cystoscopy and samples were analyzed blinded to the clinical status of the participants. Means (±SD) and receiver operating characteristic (ROC) curve analysis were used to compare assay performance and to assess the diagnostic accuracy of the diagnostic signature.

**Results:**

Comparative diagnostic performance analyses revealed an AUROC value of 0.9258 for the multiplex assay and 0.9467 for the combination of the single-target ELISA assays (*p* = 0.625), so there was no loss of diagnostic utility for the MSD multiplex assay. Analysis of the independent 200-sample cohort using the multiplex assay achieved an overall diagnostic sensitivity of 0.85, specificity of 0.81, positive predictive value 0.82 and negative predictive value 0.84.

**Conclusions:**

It is technically feasible to simultaneously monitor complex urinary diagnostic signatures in a single assay without loss of performance. The described protein-based assay has the potential to be developed for the non-invasive detection of BCa.

## Background

Urine based assays that can non-invasively detect bladder cancer (BCa) have the potential to improve the diagnosis of BCa and help to avoid unnecessary and invasive diagnostic procedures. As such, several urine-based commercial molecular tests have been FDA-approved for BCa detection and surveillance. These tests include the measurement of soluble proteins such as bladder tumor antigen (BTA) [1], and nuclear matrix protein 22 (NMP22) [2, 3], proteins detected on fixed urothelial cells (ImmunoCyt) [4], and chromosomal aberrations detected by fluorescent in situ hybridization (Urovysion) [5]. Because of their marginal detection performance, these urine-based assays have a limited role in the management of patients at risk for BCa, thus, the search for non-invasive urine-based tests with clinical utility for BCa continues.

The advent of advanced molecular profiling techniques has enabled the derivation of molecular signatures that hold promise for more accurate and individualized patient evaluation [6]. A number of molecular signature assays are now being incorporated into clinical practice [7, 8], but the assays employed to monitor multiple targets per sample are, to date, rather complex and thus, expensive, and often require centralized processing and analysis.

We have previously coupled high throughput, discovery-based technology (i.e., genomics and proteomics) with bioinformatics in order to derive diagnostic signatures that show promise for the accurate detection of BCa in voided urine samples [9–12]. Integration of data and selection based on *p* value, fold change and availability of antibodies resulted in a 14-protein biomarker panel for subsequent testing and refinement in independent cohorts. Using commercial ELISA assay kits directed at the biomarker panel, we performed three independent experiments. First, we analyzed voided urines from 127 subjects (64 with BCa and 63 controls) and confirmed that 10 of the 14 biomarkers were significantly altered in BCa compared to controls [13–16]. Next, we reported the validation of the 10-biomarker diagnostic panel (IL8, MMP9, MMP10, SERPINA1, VEGFA, ANG, CA9, APOE, SDC1 and SERPINE1) in a large cohort of patients (n = 308; 102 BCa and 206 controls) including controls with diverse urologic conditions (e.g., urolithiasis, moderate-severe voiding symptoms, urinary tract infection and hematuria) [17]. Recently, an outside laboratory externally validated the 10-biomarker diagnostic panel in a large cohort of patients (n = 320; 183 BCa and 137 controls) [18]. In this study, we investigated the feasibility of developing a multiplex assay that could accurately and simultaneously monitor the diagnostic biomarkers in an efficient format for potential clinical application. A custom multiplex assay, using MULTI-ARRAY^®^ technology (Meso Scale Diagnostics, LLC), was constructed and the analytical performance was compared with data obtained from individual ELISA assays directed at each of the same ten urinary proteins.

The multiplex assay was then used to evaluate the diagnostic signature in an independent cohort to determine sensitivity, specificity, positive predictive value (PPV) and negative predictive value (NPV).

## Methods

### Patients and specimen processing

Under Western Institutional Review Board approval (IRB #Rosser 2014-1), previously collected and banked voided urine samples were available for analysis. Voided urine samples were collected prior to cystoscopy in all BCa subjects and controls and samples were analyzed blinded to the clinical status of the participants, thus the study satisfies both PRoBE and STARD study design [19, 20]. Patients with known renal disease or documented renal insufficiency were excluded from the current study. The study consisted of two independent cohorts (Table 1); cohort #1 consisted of 62 subjects (29 with newly diagnosed BCa and 33 with no previous history of urothelia carcinoma, gross hematuria, active urinary tract infection or urolithiasis, i.e., controls) and cohort #2 consisted of 200 subjects (100 with newly diagnosed BCa and 100 with no previous history of urothelia carcinoma, gross hematuria, active urinary tract infection or urolithiasis, i.e., controls). Controls for the two cohorts consisted of healthy volunteers and individuals with voiding symptoms or microscopic hematuria. All 62 subjects in cohort #1 had their urines analyzed by individual commercial ELISA kits directed towards the ten targets in addition to the MSD multiplex assays in order to compare and contrast the two diagnostic modalities. Cohort #2 was only analyzed by the multiplex assay. Clinical information associated with these urine samples were queried from our database.

**Table 1:** Demographic and clinical-pathologic characteristics of study cohorts

	Cohort #1		P value*	Cohort #2		P value*
	Bladder cancer^a^ (n = 29)	Benign^b^ and healthy controls (n = 33)		Bladder cancer^a^ (n = 100)	Benign^b^ and healthy controls (n = 100)	
Median age (range, years)	68 (51, 93)	50 (20, 81)	<0.0001	70 (20, 89)	50.5 (18, 81)	<0.0001
Male:female ratio	25:4	27:6	0.639	81:18	81:19	0.882
Clinical stage and grade						
Tis high-grade	3 (10.3 %)			4 (4.0 %)		
Ta low-grade	4 (13.8 %)			17 (17.0 %)		
Ta high-grade	6 (20.7 %)			11 (11.0 %)		
T1 low-grade	0 (0 %)			4 (4.0 %)		
T1 high-grade	3 (10.3 %)			22 (22.0 %)		
≥T2 high-grade	13 (44.8 %)			42 (42.0 %)		

* Wilcoxon rank sum test

^a^Primary BCa; no patient with a history of BCa

^b^Voiding symptoms, microscopic hematuria

Each urine sample was centrifuged at 600×*g* 4 °C for 5 min. The supernatant was decanted and aliquoted, while the urinary pellet was snap frozen. Both the supernatant and pellet were stored at −80 °C prior to analysis. Aliquots of urine supernatants were thawed and analyzed for protein content using a Pierce 660-nm Protein Assay Kit (Thermo Fisher Scientific Inc., Waltham, MA, USA). Frozen aliquots of urine samples were thawed and protein content was measured using a Pierce 660-nm Protein Assay Kit (Thermo Fisher Scientific Inc., Waltham, MA, USA) and a microplate reader (Synergy HT, BioTek Instruments, Winooski, VT, USA). The relatively constant production of creatinine, a non-enzymatically metabolite of creatine, makes urinary creatinine a useful tool for normalizing the levels of other molecules found in urine [21]. The concentration of urinary creatinine was measured using a commercially available enzymatic assay (Cat # KGE005 R&D Systems Inc., Minneapolis, MN, USA) according to the manufacturer’s instructions. Briefly, urine supernatants were treated with alkaline picrate solution, which when creatinine is present, yields an orange-red color. Intensity at 490 nm corresponds to the concentration of creatinine in the sample. Creatinine concentrations of unknown samples were calculated by comparison to a standard curve.

### Commercial enzyme-linked immunosorbent assays (ELISA)

Levels of human Interleukin 8 (IL8, Cat # ab46032 Abcam), Matrix Metalloproteinase 9 (MMP9, Cat # DMP900 R&D Systems Inc.), Plasminogen Activator Inhibitor 1 (SERPINE1, Cat # EA-0207 Signosis Inc.), Vascular Endothelial Growth Factor A (VEGFA, Cat # 100663 Abcam), Angiogenin (ANG, Cat # CK400 CellSciences), Carbonic Anhydrase 9 (CA9, Cat # DCA900 R&D Systems Inc.), Matrix Metalloproteinase 10 (MMP10, Cat # DMP1000 R&D Systems Inc.), Apolipoprotein E (APOE, Cat # KA 1031 Abnova), Syndecan 1 (SDC1, Cat # ab46506 Abcam) and A1AT (SERPINA1, Cat # ab108799, Abcam) were monitored in urine samples using commercial enzyme-linked immunosorbent assays (ELISA) as previously reported [17]. Calibration curves were prepared using purified standards for each protein assessed. Curve fitting was accomplished by either linear or four-parameter logistic regression following the manufacturer’s instructions.

### MULTI-ARRAY assay

A panel of commercially available monoclonal antibodies against each of the ten biomarkers was screened pairwise and antibody pairs were selected from an unbiased screen using recombinant protein and normal pooled urine. The final monoclonal antibody pair (capture and detection) was selected based on sensitivity, specificity, physical properties, and recognition of native protein. Detection monoclonal antibodies were labeled with ruthenium (SULFO-TAG™ NHS-Ester), according the manufacturer’ s instructions (Meso Scale Diagnostics, LLC, Rockville, MD, USA). The multiplex assay, using MULTI-SPOT^®^ plates, is based on a proprietary combination of electrochemiluminescence detection and patterned arrays [22]. Briefly, immobilized capture antibodies were placed in 350–500 μm spots at the bottom of polypropylene 96-well plates to capture target proteins (for details see; http://www.mesoscale.com/CatalogSystemWeb/WebRoot/). Each spot is coated with a different analyte capture antibody, in this case against IL8, MMP9, MMP10, ANG, APOE, SDC1, A1AT, PAI1, CA9 and VEGFA. The sandwich immunoassay complex that forms generates light when the instrument applies a voltage. Optics and a cooled-CCD camera then collect and quantitatively measure light emitted and processing algorithms convert the data into target concentrations. A seven point standard curve across the 4 log dynamic range of the assays was included in the current assay design.

Initial sample testing noted the need to dilute 7 of the 10 biomarkers fourfold (IL8, MMP9, MMP10, APOE, PAI1, CA9 and VEGFA) and 3 of the 10 biomarkers 200-fold (ANG, SDC1, A1AT) to ensure concentrations were within optimal range on the calibration curve. Thus, we elected to divide the ten biomarkers into two patterned array panels (Panel 1—IL8, MMP9, MMP10, VEGFA, CA9, APOE and PAI1 and Panel 2—A1AT, ANG and SDC1.

Urine samples were handled on ice and diluted with MSD Assay Diluent 37, designed to reduce the effects from heterophilic antibodies and other interferents. Samples and standards (50 μl) were loaded onto the MSD^®^ plate in duplicate with a multichannel pipettor in order to reduce pipetting error and allowed to incubate for 2 h and then washed out. SULFO-TAG conjugated detection antibody (25 μl) was added to each well and allowed to incubate for 2 h and then washed out. Subsequently, the sandwich immunoassay complex that forms was incubated MSD Read Buffer (150 μl), the electrochemiluminescent substrate, and levels of electrochemiluminescent units were measured on the QuickPlex^®^ SQ 120 (MSD) instrument. Standard curves were constructed using MSD Discovery Workbench^®^ 4.0, which allows for the selection of multiple non-linear and linear equations to fit the standard curve. Optimal curve fits were determined by visual graph evaluation and comparison of Akaike’s information criteria (AIC) values [23].

### Data analysis

First, urinary concentrations of each of the ten biomarkers were normalized using urinary creatinine concentration. Next, we compared the diagnostic ability associated with the combination of the individual commercial ELISA assays directed at our ten biomarkers versus the diagnostic ability of the multiplex assay, which encompassed these same ten biomarkers. Then we investigated the diagnostic performance of the multiplex assay in an independent cohort. Logistic regression analysis with BCa status (yes vs. no) as the response variable and the ten biomarkers as the explanatory variables was performed. The individual biomarkers were combined into a linear combination with the regression coefficients obtained in logistic regression as the weights, and the linear combination was used as a combined score for the detection of BCa. Cutoff thresholds were identified in cohort #1 and applied to the analysis of cohort #2. Then for a given cutoff threshold, we calculated the sensitivity and specificity of the test. We generated a ROC curve by plotting values for sensitivity against the false-positive rates (1-specificity) for various cutoff thresholds [24]. The relative ability of the combination of biomarkers to indicate BCa was estimated by calculating the area under the ROC curves (AUC), with a higher AUC indicating a stronger predictor. We select the optimal cutoff value defined by the Youden index [25], i.e., the cutoff value that maximizes the sum of the sensitivity and the specificity. We estimated the sensitivity, specificity, PPV and NPV of the combination of biomarkers at the optimal cutoff value. Statistical significance in this study was set at *p* < 0.05 and all reported *p* values were 2-sided. All analyses were performed using SAS software version 9.3 (SAS Institute Inc., Cary, NC, USA).

## Results

### Multiplex assay characterization

The physical components, a library of capture and detection antibodies, and the secondary reagents for the MULTI-ARRAY technology, have undergone extensive optimization by the manufacturers for consistent implementation. Ranges for each analyte assay were evaluated by dilution of standards to determine upper ranges where high-end hook effect and apparent antibody saturation are avoided and lower ranges that are above detection limits. Lower limits of detection (LLOD) were calculated based on 2.5× the standard deviation of the background assessed across the plates run during sample testing. LLOD for each analyte in Panel 1 was 0.081 pg/ml IL8, 38.4 pg/ml MMP9, 3.39 pg/ml MMP10, 0.030 pg/ml VEGFA, 2.13 pg/ml CA9, 236 pg/ml APOE and 1.97 pg/ml PAI1 and in Panel 2 was 17.4 pg/ml A1AT, 0.13 pg/ml ANG and 0.62 pg/ml SDC1, demonstrating sufficient sensitivity to detect proteins present in small amounts in voided urine samples. Intra assay precision was measured with acceptance criteria of a coefficient of variation (% CV) of less than 15.0. Median inter assay variability across all plates was also determined to be less than 15 % CV for each analyte. As the technology is an array, all components were checked for cross reactivity with other components in the antigen and antibody cocktails and confirmed to have less than 0.5 % cross reactivity. Lower limits of quantification (LLOQ) were estimated as being at the concentration with signals least three times over background and the lowest point of the 7-point standard curve where the back-fit regression recovered to within 20 % of the known value and had % CVs of less than 20 %. The dilution linearity of five pooled urine samples was between 80 and 120 % for all analytes at the recommended sample dilution factor, suggesting that there was not any interference due to the matrix.

### Multiplex assay vs. individual ELISA assays

**Fig. 1:**
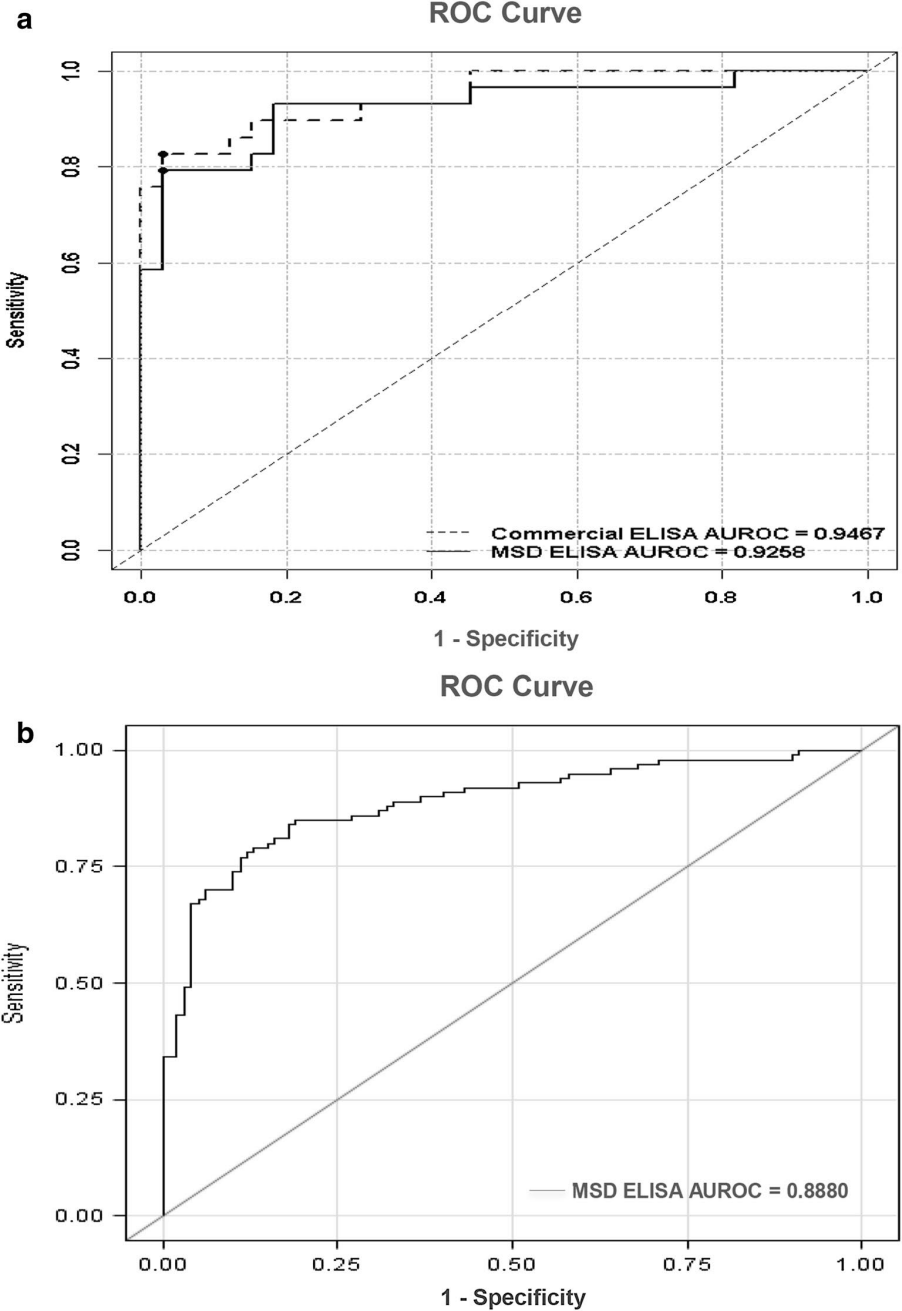
Diagnostic performance of bladder cancer-associated molecular panel comprised of 10 biomarkers. **a** ROC curves were plotted to compare the performance characteristics of the MULTI-ARRAY^®^ Assay (*solid line*) and the combined individual commercial ELISA assays (*dotted line*) in cohort #1 (62 subjects). Based on the area under the ROC curve (AUROC), Youden Index cutoff values that maximized the sum of sensitivity and specificity were determined for the combination of biomarkers. The MULTI-ARRAY^®^ Assay achieved an overall sensitivity of 0.79 and specificity of 0.97 (AUROC 0.9258). The combination of data from the individual target ELISA assays achieved an overall sensitivity of 0.82 and specificity of 0.97 (AUROC 0.9467). **b** ROC curve to illustrate the performance characteristics of the MULTI-ARRAY^®^ Assay in cohort #2 (200 subjects). The MULTI-ARRAY^®^ assay achieved an overall sensitivity of 0.85 and specificity of 0.81 (AUROC 0.8880)

In order to test the robustness of the multiplex assay, 62 urines samples (29 from BCa subjects) were monitored for the ten biomarkers using both the multiplex assay and the ten commercially available individual assays. Demographics and disease characteristics of cohort #1 are summarized in Table 1. Urinary concentrations of 7 of the 10 biomarkers were significantly elevated in patients with BCa compared to controls in both multiplex assay and individual ELISA assays (Table 2). A combinatorial analysis of all ten biomarkers using optimal cutoff values defined by Youden index calculations resulted in an AUROC (Fig. 1a) of 0.9258 (95 % CI 0.8559–0.9958) in the multiplex assay and 0.9467 (95 % CI 0.8944–0.9990) for the individual ELISA assays (*p* = 0.625). Thus, there was no difference between the diagnostic performances of the two formats. The multiplex assay achieved an overall sensitivity of 0.79, specificity of 0.97, PPV of 0.96 and NPV of 0.84 for BCa classification. Table 3 provides AUROC for each biomarker and combination of the ten biomarkers tested in the multiplex assay and individual ELISA assay.

**Table 2:** Mean urinary (±SD) concentrations of biomarkers assessed by Multi-Array and commercial ELISA assays in cohort #1

	Multi-Array^®^			Commercial ELISA		
	Total bladder cancer (n = 29)	Total controls (n = 33)	*p* value	Total bladder cancer (n = 29)	Total controls (n = 33)	*p* value
IL8 (pg/ml)	1429.3 ± 2897.7	123.1 ± 510.2	<0.0001	1155.7 ± 2179.7	33.3 ± 45.1	<0.0001
MMP9 (ng/ml)	24.4 ± 64.2	3.2 ± 10.6	<0.0001	4.8 ± 4.1	0.9 ± 1.4	<0.00001
A1AT (ng/ml)	4968.0 ± 8965.9	412.9 ± 513.9	0.0004	2362.2 ± 3682.4	218.8 ± 593.6	<0.0001
ANG (pg/ml)	8487.6 ± 25,979.1	816.3 ± 947.4	0.004	538.5 ± 863.9	174.0 ± 210.8	0.016
VEGFA (pg/ml)	357.7 ± 437.6	306.7 ± 467.9	0.481	294.5 ± 795.4	166.3 ± 137.5	0.631
CA9 (pg/ml)	35.9 ± 63.6	15.4 ± 22.6	0.828	2.3 ± 0.0	2.3 ± 0.0	1.000
MMP10 (pg/ml)	112.7 ± 196.9	26.1 ± 64.8	0.002	12.2 ± 43.7	4.1 ± 0.0	0.301
APOE (pg/ml)	210,368.9 ± 758,136.6	7316.1 ± 11,258.8	0.016	46,241.4 ± 38,121.7	30,000.0 ± 0.0	0.001
PAI1 (ng/ml)	5.2 ± 13.7	0.2 ± 1.0	<0.0001	0.1 ± 0.1	0.1 ± 0.0	0.065
SDC1 (pg/ml)	10,393.9 ± 12,279.5	13,477.3 ± 10,745.7	0.204	53,545.5 ± 98,390.1	103,803.3 ± 108,345.5	0.0001

**Table 3:** Comparison of bladder cancer diagnostic biomarker performance in Multi-Array ^®^ assay and commercial assays in cohort #1

Biomarker	Area under the curve of Multi-Array assay	Area under the curve of commercial assays	Difference		P value
			Commercial-Multi-Array	95 % confidence interval	
IL-8	0.8673	0.9018	0.0345	(−0.0534, 0.1224)	0.442
MMP-9	0.8474	0.8443	−0.0031	(−0.0673, 0.0611)	0.924
A1AT	0.8443	0.8694	0.0251	(−0.0541, 0.1043)	0.535
Angiogenin	0.8109	0.7555	−0.0554	(−0.1503, 0.0395)	0.253
VEGF	0.6897	0.6228	−0.0669	(−0.2323, 0.0986)	0.428
CA-9	0.5705	0.6468	0.0763	(−0.0886, 0.2411)	0.364
MMP-10	0.7753	0.6792	−0.0961	(−0.2315, 0.0392)	0.164
APOE	0.7827	0.7043	−0.0784	(−0.2338, 0.0771)	0.323
PAI-1	0.8924	0.6740	−0.2184	(−0.3506, −0.0862)	0.001
Syndecan	0.5831	0.6301	0.0470	(−0.2118, 0.3058)	0.722
All 10 biomarkers	0.9258	0.9467	0.0209	(−0.0628, 0.1046)	0.625

### Validation of the multiplex assay

In order to validate the diagnostic performance of the assay in a larger cohort, the multiplex assay was applied to an independent cohort of 200 urine samples (100 from BCa subjects). Demographics and disease characteristics of the cohort (cohort #2) are summarized in Table 1. In this validation cohort, urinary concentrations of 9 of the 10 biomarkers were significantly elevated in patients with BCa compared to controls (Table 4). Furthermore, the urinary concentrations of 3 of the 10 biomarkers (IL8, MMP10 and PAI1) were significantly elevated in patients with high-grade BCa compared to low-grade BCa, while 7 of the 10 biomarkers (IL8, MMP9, A1AT, ANG, VEGF, MMP10 and PAI1) were significantly elevated in patients with muscle invasive BCa (MIBC) compared to non-muscle invasive BCa (NMIBC) (Table 4). Based on our prediction rule developed from cohort #1, the multiplex assay achieved an overall diagnostic sensitivity of 0.85 %, specificity of 0.81 %, PPV 0.82 % and NPV 0.84 % for the combination of the ten biomarkers (Fig. 1b). Table 5 provides AUROC and corresponding sensitivity, specificity, PPV and NPV values for all biomarkers tested.

**Table 4:** Mean urinary (±SD) concentrations and clinical associations of 10 biomarkers in cohort #2 assessed by Multi-Array assay

	Total bladder cancer (50 %), n = 100	Low-grade bladder cancer (21 %), n = 21	High-grade bladder cancer (79 %), n = 79	NMIBC (58 %), n = 58	MIBC (42 %), n = 42	Total controls (50 %), n = 100
IL8 (pg/ml)*^,^^^, +^	761.6 ± 804.4	348.3 ± 541.8	871.5 ± 829.4	593.2 ± 766.8	994.3 ± 805.9	122.8 ± 380.1
MMP9 (ng/ml)*^,^^+^	115.7 ± 392.9	26.0 ± 61.0	139.6 ± 438.4	89.0 ± 362.1	152.6 ± 433.6	2.8 ± 11.7
A1AT (ng/ml)*^,^^+^	4002.6 ± 7384.7	2961.5 ± 7204.7	4279.4 ± 7452.3	3949.8 ± 8302.9	4075.6 ± 5985.6	993.6 ± 2962.6
ANG (pg/ml)*^,^^+^	6501.0 ± 23,967.9	10,109.0 ± 42,456.3	5541.9 ± 16,201.7	6567.1 ± 27,404.6	6409.7 ± 18,520.3	619.0 ± 1093.1
VEGFA (pg/ml)*^,^^+^	1321.0 ± 1969.9	971.0 ± 1747.0	1414.1 ± 2025.0	1082.0 ± 1832.9	1651.1 ± 2122.9	570.4 ± 586.8
CA9 (pg/ml)*	168.6 ± 461.9	126.9 ± 398.6	179.7 ± 479.0	142.9 ± 409.9	204.1 ± 528.6	1.0 ± 0.0
MMP10 (pg/ml)*^,^^^,^^+^	2576.3 ± 12,512.1	977.4 ± 3490.8	3001.3 ± 13,953.7	1444.5 ± 5444.0	4139.2 ± 18,234.5	5.0 ± 0.0
APOE (pg/ml)*	199,003.5 ± 587,474.7	202,108.4 ± 695,029.2	198,178.2 ± 560,516.7	266,463.1 ± 751,668.8	105,845.1 ± 180,362.0	21,654.7 ± 37,121.9
PAI1 (ng/ml)*^,^^^,^^+^	26.2 ± 53.7	12.4 ± 43.8	29.9 ± 55.7	17.6 ± 47.8	38.1 ± 59.5	0.7 ± 3.8
SDC1 (pg/ml)	14,853.3 ± 43,824.9	9958.5 ± 9625.1	16,154.4 ± 49,048.9	10,814.6 ± 15,586.0	20,430.5 ± 65,153.2	10,459.7 ± 14,248.5

*NMIBC* non-muscle invasive bladder cancer, *MIBC* muscle invasive bladder cancer

* *P* < 0.05 comparing total bladder cancer to total controls

^^^ *P* < 0.05 comparing low-grade bladder cancer to high-grade bladder cancer

^+^ *P* < 0.05 comparing NMIBC to MIBC

**Table 5:** Biomarker performance of Multi-Array ^®^ assay for bladder cancer detection in cohort #2

Biomarker	Area under the curve	95 % confidence interval	No. of correctly predicted events	No. of correctly predicted nonevents	No. of nonevents predicted as events	No. of events predicted as nonevents	Sen. (%)	Spec. (%)	PPV (%)	NPV (%)
IL8	0.8489	0.7948–0.9030	74	82	18	26	74.0	82.0	80.4	75.9
MMP9	0.7620	0.6971–0.8269	70	69	31	30	70.0	69.0	69.3	69.7
A1AT	0.7935	0.7316–0.8554	72	78	22	28	72.0	78.0	76.6	73.6
ANG	0.7603	0.6949–0.8257	45	95	5	55	45.0	95.0	90.0	63.3
VEGFA	0.7121	0.6412–0.7830	57	76	24	43	57.0	76.0	70.4	63.9
CA9	0.6885	0.6159–0.7611	53	77	23	47	53.0	77.0	69.7	62.1
MMP10	0.7507	0.6844–0.8170	50	88	12	50	50.0	88.0	80.6	63.8
APOE	0.7240	0.6543–0.7937	52	84	16	48	52.0	84.0	76.5	63.6
PAI1	0.8335	0.8173–0.9097	74	86	13	24	74.0	86.0	85.4	76.8
SDC1	0.6540	0.5787–0.7292	85	41	59	15	85.0	41.0	59.0	73.2
10-biomarker combination	0.8880	0.8416–0.9344	85	81	19	15	85.0	81.0	81.7	84.4

## Discussion

The data presented here demonstrates for the first time a multiplex immunoassay assay capable of measuring a unique diagnostic signature in voided urine samples from patients with BCa. The custom-designed multiplex assay showed excellent limits of detection in the low pg/ml range and wide dynamic ranges up to at least 5000 pg/ml. Specifically, each of the biomarkers analyzed (i.e., IL8, MMP9, MMP10, VEGFA, CA9, APOE, PAI1, A1AT and ANG) were present at higher levels in voided urines from BCa subjects compared to controls. The only biomarker that was not consistently elevated in the urines of BCa patients was SDC1. We have previously reported that while SDC1 is not always elevated in BCa urine samples relative to controls it can provide prognostic information based on its association with tumor grade and stage [26]. Though not statistically significant, mean values of SDC1 were 60 % higher when comparing high-grade to low-grade tumors and 110 % higher when comparing MIBC to NMIBC in this study.

We have reported the derivation and the validation of the urinary 10-biomarker BCa diagnostic signature in a number of studies with a total of over 800 subjects [13–18]. Recent work from our laboratory using quantitative immunohistochemical staining techniques for the detection of the signature proteins has shown a strong association of expression and malignancy in human bladder tumor tissues [27]. Taken together, these data are the basis for a phased, methodical course to develop a robust multiplex diagnostic assay to assist in the non-invasive detection of BCa.

There is a growing demand for the integration of multiplex molecular signatures into single assays in order to obtain favorable assay properties such as reduced sample volume, decreased processing time, low cost analysis and low reagent consumption. Several multiplex protein measurement services are available (e.g., Sample Testing Services of Quansys Biosciences Inc.—microplate-based; Aushon Biosystems and SearchLight Assays Services—microplate-based; Milliplex MAP—bead-based; and RayBiotech, Inc.—slide-based), and several studies have reported that multiplex ELISA procedures appear suitable and reliable for tissue lysate and serum [28, 29]. While there are inherent, usually subtle differences between the various multiplex technologies, the overall approach and goals are the same; to construct a rapid, cost effective, and reliable immunoassay. The reliability of any immunoassay assay is due in part to the characteristics of specific capture and detection antibody pairs that are employed for the measurement of a specific protein. In particular, the PAI1 antibody pair used in the MULTI-ARRAY assay plate performed significantly better than the pair in the commercial ELISA kit (AUROC—0.8924 vs. 0.6740, respectively, *p* = 0.001). For inclusion in multiplex assays, selection of antibody pairs is driven by specificity for the target, but it also important that there should be no cross-reactivity with other proteins or antibodies in the assay milieu.

The ability of each of the test biomarkers included in the multiplex assay to predict the presence of BCa was analyzed using nonparametric ROC analyses. Urinary PAI1 was the most accurate single biomarker with an AUROC of 0.8335 (95 % CI 0.8173–0.9097), a sensitivity of 74 %, specificity of 86 %, PPV of 85 % and NPV of 77 %, followed closely by urinary IL8—AUROC of 0.8489 (Table 5). Many of the single biomarkers achieved respectable performance values, but the power of the multiplex approach is revealed when the data are combined using a mathematical rule. The combination of data from all ten biomarkers measured on the multiplex assay achieved an AUROC of 0.8880 (95 % CI 0.8416–0.9344), with an overall sensitivity of 0.85, specificity of 0.81, PPV of 0.82 and NPV of 0.84 for BCa classification. The combinatorial power achieved using a biomarker panel approach attests to the heterogeneity of bladder lesions within the sampled population, and the importance of signatures that can overcome this heterogeneity. From a practical point of view, multiplex assays may also reduce the need for repeat sample testing due to more robust scoring systems.

The performance values achieved by the described multiplex assay far exceed those achieved by voided urine cytology or single biomarker tests for BCa detection, but as we refine and optimize the technical aspects of the assay and analyze additional samples, we expect to be able to further improve considerably assay performance. Using 2002 Medicare data, we noted the unit cost for testing a voided urine cytology (VUC) sample of $50.71 to be approximate ($55) to the cost of our multiplex assay. The benefits of the multiplex assay include (a) ease of performance and (b) rapidity of obtaining results. We are proposing a prospective study comparing head-to-head VUC and our multiplex assay.

Clinically, accurate non-invasive BCa assays would have a clear impact on the clinical management of patients with BCa. The ultimate goal is to be able to detect BCa in a timely manner such that the patient can expect an improved survival as well as improved quality of life. For clinical implementation, a molecular test needs to be cost-effective, as well as accurate, especially if that test is to be used over a long period of follow-up as in the case for BCa. The detection of urinary proteins through multiplexed analyses has the potential to be relatively simple to perform and interpret, and affordable. We feel that the MSD multiplex assay described here is robust enough to deserve continued clinical development and to be the focus of a large prospective multiple center study.

## Conclusion

Based on these encouraging preliminary data, we believe that the MSD multiplex assay for the non-invasive detection of BCa is a viable new platform that can be developed to be a simple, yet accurate diagnostic tool. Importantly, the assay has very sensitive levels of detection for the simultaneous monitoring of multiple protein targets yet can be readily implemented into a CLIA certified laboratory setting.

## References

[1] Thomas L, Leyh H, Marberger M, Bombardieri E, Bassi P, Pagano F, Pansadoro V, Sternberg CN, Boccon-Gibod L, Ravery V, Le Guludec D, Meulemans A, Conort P, Ishak L (1999). Multicenter trial of the quantitative BTA TRAK assay in the detection of bladder cancer. Clin Chem.

[2] Grossman HB, Soloway M, Messing E, Katz G, Stein B, Kassabian V, Shen Y (2006). Surveillance for recurrent bladder cancer using a point-of-care proteomic assay. JAMA.

[3] Grossman HB, Messing E, Soloway M, Tomera K, Katz G, Berger Y, Shen Y (2005). Detection of bladder cancer using a point-of-care proteomic assay. JAMA.

[4] Mian C, Pycha A, Wiener H, Haitel A, Lodde M, Marberger M (1999). Immunocyt: a new tool for detecting transitional cell cancer of the urinary tract. J Urol.

[5] Sarosdy MF, Schellhammer P, Bokinsky G, Kahn P, Chao R, Yore L, Zadra J, Burzon D, Osher G, Bridge JA, Anderson S, Johansson SL, Lieber M, Soloway M, Flom K (2002). Clinical evaluation of a multi-target fluorescent in situ hybridization assay for detection of bladder cancer. J Urol.

[6] Diamandis EP (2012). The failure of protein cancer biomarkers to reach the clinic: why, and what can be done to address the problem?. BMC Med.

[7] Nguyen B, Cusumano PG, Deck K, Kerlin D, Garcia AA, Barone JL, Rivera E, Yao K, de Snoo FA, van den Akker J, Stork-Sloots L, Generali D (2012). Comparison of molecular subtyping with BluePrint, MammaPrint, and TargetPrint to local clinical subtyping in breast cancer patients. Ann Surg Oncol.

[8] Malo TL, Lipkus I, Wilson T, Han HS, Acs G, Vadaparampil ST (2012). Treatment choices based on OncotypeDx in the breast oncology care setting. J Cancer Epidemiol.

[9] Yang N, Feng S, Shedden K, Xie X, Liu Y, Rosser CJ (2011). Urinary glycoprotein biomarker discovery for bladder cancer detection using LC/MS–MS and label-free quantification. Clin Cancer Res.

[10] Kreunin P, Zhao J, Rosser C, Urquidi V, Lubman DM, Goodison S (2007). Bladder cancer associated glycoprotein signatures revealed by urinary proteomic profiling. J Proteome Res.

[11] Urquidi V, Goodison S, Cai Y, Sun Y, Rosser CJ (2012). A candidate molecular biomarker panel for the detection of bladder cancer. Cancer Epidemiol Biomark Prev.

[12] Rosser CJ, Liu L, Sun Y, Villicana P, McCullers M, Porvasnik S (2009). Bladder cancer-associated gene expression signatures identified by profiling of exfoliated urothelia. Cancer Epidemiol Biomark Prev.

[13] Urquidi V, Goodison S, Ross S, Chang M, Dai Y, Rosser CJ (2012). Diagnostic potential of urinary <alpha> 1-antitrypsin and apolipoprotein E in the detection of bladder cancer. J Urol.

[14] Urquidi V, Kim J, Chang M, Dai Y, Rosser CJ, Goodison S (2012). CCL18 in a multiplex urine-based assay for the detection of bladder cancer. PLoS One.

[15] Urquidi V, Chang M, Dai Y, Kim J, Wolfson ED, Goodison S (2012). IL-8 as a urinary biomarker for the detection of bladder cancer. BMC Urol.

[16] Urquidi V, Goodison S, Kim J, Chang M, Dai Y, Rosser CJ (2012). Vascular endothelial growth factor, carbonic anhydrase 9, and angiogenin as urinary biomarkers for bladder cancer detection. Urology.

[17] Goodison S, Chang M, Dai Y, Urquidi V, Rosser CJ (2012). A multi-analyte assay for the non-invasive detection of bladder cancer. PLoS One..

[18] Chen LM, Chang M, Dai Y, Chai KX, Dyrskjøt L, Sanchez-Carbayo M, Szarvas T, Zwarthoff EC, Lokeshwar V, Jeronimo C, Parker AS, Ross S, Borre M, Orntoft TF, Jaeger T, Beukers W, Lopez LE, Henrique R, Young PR, Urquidi V, Goodison S, Rosser CJ (2014). External validation of a multiplex urinary protein panel for the detection of bladder cancer in a multicenter cohort. Cancer Epidemiol Biomark Prev.

[19] Feng Z, Kagan J, Pepe M, Thornquist M, Ann Rinaudo J, Dahlgren J, Krueger K, Zheng Y, Patriotis C, Huang Y, Sorbara L, Thompson I, Srivastava S (2013). The early detection research network’s specimen reference sets: paving the way for rapid evaluation of potential biomarkers. Clin Chem.

[20] Bossuyt PM, Reitsma JB, Bruns DE, Gatsonis CA, Glaszious PP, Irwig LM, Lijmer JG, Moher D, Rennie D, de Vet HC, STARD Group (2004). Towards complete and accurate reporting of studies of diagnostic accuracy: the STARD initiative. Fam Pract.

[21] Reid CN, Stevenson M, Abogunrin F, Ruddock MW, Emmert-Streib F, Lamont JV (2012). Standardization of diagnostic biomarker concentrations in urine: the hematuria caveat. PLoS One.

[22] O’Shannessy DJ, Somers EB, Palmer LM, Thiel RP, Oberoi P, Heath R, Marcucci L (2013). Serum folate receptor alpha, mesothelin and megakaryocyte potentiating factor in ovarian cancer: association to disease stage and grade and comparison to CA125 and HE4. J Ovarian Res.

[23] Motulsky H, Christopoulos H (2004). Fitting models to biological data using linear and nonlinear regression: a practical guide to curve fitting.

[24] Edgar R, Domrachev M, Lash AE (2002). Gene expression omnibus: NCBI gene expression and hybridization array data repository. Nucleic Acids Res.

[25] Fluss R, Faraggi D, Reiser B (2005). Estimation of the Youden Index and its associated cutoff point. Biom J.

[26] Miyake M, Lawton A, Dai Y, Chang M, Mengual L, Alcaraz A, Goodison S, Rosser CJ (2014). Clinical implications in the shift of syndecan-1 expression from the cell membrane to the cytoplasm in bladder cancer. BMC Cancer.

[27] Zhang G, Gomes-Giacoia E, Dai Y, Lawton A, Miyake M, Furuya H, Goodison S, Rosser CJ (2014). Validation and clinicopathologic associations of a urine-based bladder cancer biomarker signature. Diagn Pathol.

[28] Trune DR, Larrain BE, Hausman FA, Kempton JB, MacArthur CJ (2011). Simultaneous measurement of multiple ear proteins with multiplex ELISA assays. Hear Res.

[29] Zeng L, Liu J, Wang Y, Wang L, Weng S, Chen S, Yang GY (2013). Cocktail blood biomarkers: prediction of clinical outcomes in patients with acute ischemic stroke. Eur Neurol.

